# Prospective Comparative Cohort Study of the Initial Learning Curve of Hugo^TM^ RAS for Inguinal Hernia Repair by a Proficient da Vinci Robotic Hernia Surgeon: The SUSHI Study

**DOI:** 10.3389/jaws.2025.15214

**Published:** 2025-10-16

**Authors:** Rianne Brood, Maaike Vierstraete, Dietrich De Mey, Ella Hermie, Filip Muysoms

**Affiliations:** Department of Surgery, Maria Middelares Ghent, Ghent, Belgium

**Keywords:** inguinal hernia, groin hernia, robotic surgery, laparoscopy, learning curve

## Abstract

**Background:**

Robotic-assisted surgery has become increasingly utilized in inguinal hernia repair (r-TAPP), offering enhanced precision and improved ergonomics. Differences in operative efficiency between robotic platforms during the early adoption phase are not well known. This study compares the operative times during the learning curve of r-TAPP using the Hugo^TM^ RAS and the da Vinci Xi robotic system.

**Methods:**

Patients undergoing r-TAPP with the Hugo^TM^ RAS were prospectively enrolled (Hugo SUSHI cohort). Data on patient characteristics, operation time, complication rate and quality of life scores were collected and compared to previously prospectively collected data from r-TAPP performed with the da Vinci Xi. All surgeries were performed by the same surgeon.

**Results:**

The first 50 consecutive patients operated with the Hugo^TM^ RAS (sept 2023 - dec 2024) were included in the study and compared to the first 50 patients operated with the da Vinci Xi (sept 2016 - jan 2017). Mean skin-to-skin operative time was 57.0 min with Hugo™ RAS and 62.8 min with da Vinci Xi (mean difference: 5.9 min; p = 0.09). Among the first 25 cases, skin-to-skin time was significantly shorter with Hugo™ RAS (mean difference: 11.7 min; p < 0.001), but this difference was not observed in the second 25 cases. In the Hugo SUSHI cohort two intraoperative complications occurred, neither of which were procedure related, and at 4-week follow-up 5 patients (10%) presented with an asymptomatic seroma. Postoperative quality of life was significantly improved at 4 weeks.

**Conclusion:**

For a surgeon experienced with the da Vinci platform, transition to the Hugo^TM^ RAS for r-TAPP was not associated with a measurable learning curve in terms of skin-to-skin operative time.

## Introduction

Inguinal hernias are common and a frequent indication for treatment by general surgeons. For inguinal hernias in adults a mesh-based repair is recommended for most cases [1]. Although open repair is a valid option, a minimal invasive repair either transabdominal preperitoneal (TAPP) or totally extraperitoneal repair (TEP) is favoured by many surgeons. It is recommended if resources and expertise are available [1]. Robotic-assisted laparoscopic transabdominal preperitoneal inguinal hernia repair (r-TAPP) is demonstrating rapid adoption in the United States [2–4]. Barriers to adopting this innovative technique in Europe include low availability of the robotic system to general surgeons, cost of the robotic instruments and the perception of longer operative times [5].

Until recently robotic surgery was exclusively done using the da Vinci system (Intuitive Surgical, Sunnyvale, CA, US). Getting access as a general surgeon and using robotic-assisted surgery for benign diseases like hernia surgery is challenging. Several newer robotic platforms have been marketed in recent years and since 2022 the Hugo^TM^ RAS (Medtronic, Minneapolis, MN, US) has been approved for use in general surgery, including abdominal wall surgery in Europe. Having competitive systems might increase the accessibility to perform surgeries robot assisted and might lead to a reduction in costs, making it a more affordable option. Hugo^TM^ RAS differs from the da Vinci system in that it has separate arms rather than a central boom controlling all arms and instruments. It also features an open surgeon console, unlike the immersive console of the da Vinci system, and the hand controls work differently. Previous studies have already shown that the Hugo^TM^ RAS is safe in robotic-assisted abdominal and pelvic procedures [6, 7]. An open question is how the introduction of a different robotic platform will influence efficiency and operative times for surgeons that already have reached proficiency on the da Vinci platform.

Our objective is to evaluate the evolution of the operative time during the first 50 r-TAPP procedures performed with the Hugo™ RAS by a da Vinci proficient surgeon and compare this to the operative time for r-TAPP during the same surgeon’s early adoption of the da Vinci platform. [8]. The study will also explore the efficacy of the Hugo^TM^ RAS CE approved set-up guide for inguinal hernia repair in a clinical setting and is therefore called SetUp Study for Hugo^TM^ RAS Inguinal hernia repair: the SUSHI study.

## Materials and Methods

This study is a prospective single-centre study comparing a consecutive initial series of 50 r-TAPP procedures performed with the Hugo^TM^ RAS by a proficient robotic hernia surgeon (Hugo SUSHI cohort) with a historical control group, consisting of the first 50 r-TAPP cases using the da Vinci Xi system of the same surgeon (da Vinci group) [8].

The study was approved by the ethics committee at Maria Middelares Ghent hospital with the Belgian trial number B0172023000012. The study protocol was submitted at ClinicalTrials.gov (NCT06599515) before the start of the study.

### Control Group

For the historical control group, the raw study data of a prospective cohort study on the learning curve of the da Vinci Xi system, published in 2018, were used [8]. This study was approved at Maria Middelares Ghent hospital with the Belgian trial number B300201629629. This study protocol was submitted at ClinicalTrials.gov (NCT02975401).

Inclusion criteria for both studies were almost the same except for excluding patients with a BMI higher than 35 kg/m^2^ in the da Vinci group. For this reason, in the original publication of this cohort one patient was excluded from analysis. Since we could use the raw data from the control group, we included that patient in the current analysis. No patient in the Hugo SUSHI cohort had a BMI higher than 35 kg/m^2^.

### Surgical Experience & Preparation

All procedures were performed by a single surgeon who, at the start of the SUSHI study, had over 25 years of experience with laparoscopic TAPP and more than 7 years of experience performing r-TAPP using the da Vinci Xi system. The surgeon has been a proctor for robotic surgery for 6 years having proctored 100+ hernia surgeons. At the time of the start of the da Vinci Xi, the surgeon had a 20-year experience with laparoscopic inguinal hernia repair, but no experience with robot assisted surgery.

Preparation before the first procedures with Hugo^TM^ RAS involved simulator training on the Hugo^TM^ RAS following dedicated simulator training modules and a 3-day training at the ORSI academy training centre in Melle, Belgium. This involved a day of technology training on Hugo^TM^ RAS, a day of skills acquisition on a porcine model performing cholecystectomy and a day of hernia specific training on human cadaver. This training at ORSI academy included training a second surgeon as first assist and the nursing team.

The team in the operating room consisted of a first assist and nursing team with no previous clinical experience using Hugo^TM^ RAS, but an extensive experience with the da Vinci X and da Vinci Xi systems. For the da Vinci group the nursing team had extensive experience with the da Vinci Xi system from urological robot-assisted surgery.

In the Hugo SUSHI cohort, a set-up specialist from Medtronic was present during all 50 cases, whilst in the da Vinci group logistic support from Intuitive was only present during the first 10 surgeries in the operating room.

### Participants

#### Inclusion Criteria

Adult patients scheduled for treatment of inguinal hernias with a minimally invasive surgical technique were eligible.

#### Exclusion Criteria

Excluded from participation in the study were: recurrent inguinal hernias after previous preperitoneal mesh placement, inguinal hernias after abdominal prostatectomy, patients below the age of 18 years, pregnancy, emergency surgery, absence of a signed informed consent from the patient.

#### Follow-Up

All patients were invited to attend a standard clinical outpatient follow-up visit with the surgeon at 4 weeks postoperative.

### Surgical Technique

**FIGURE 1:**
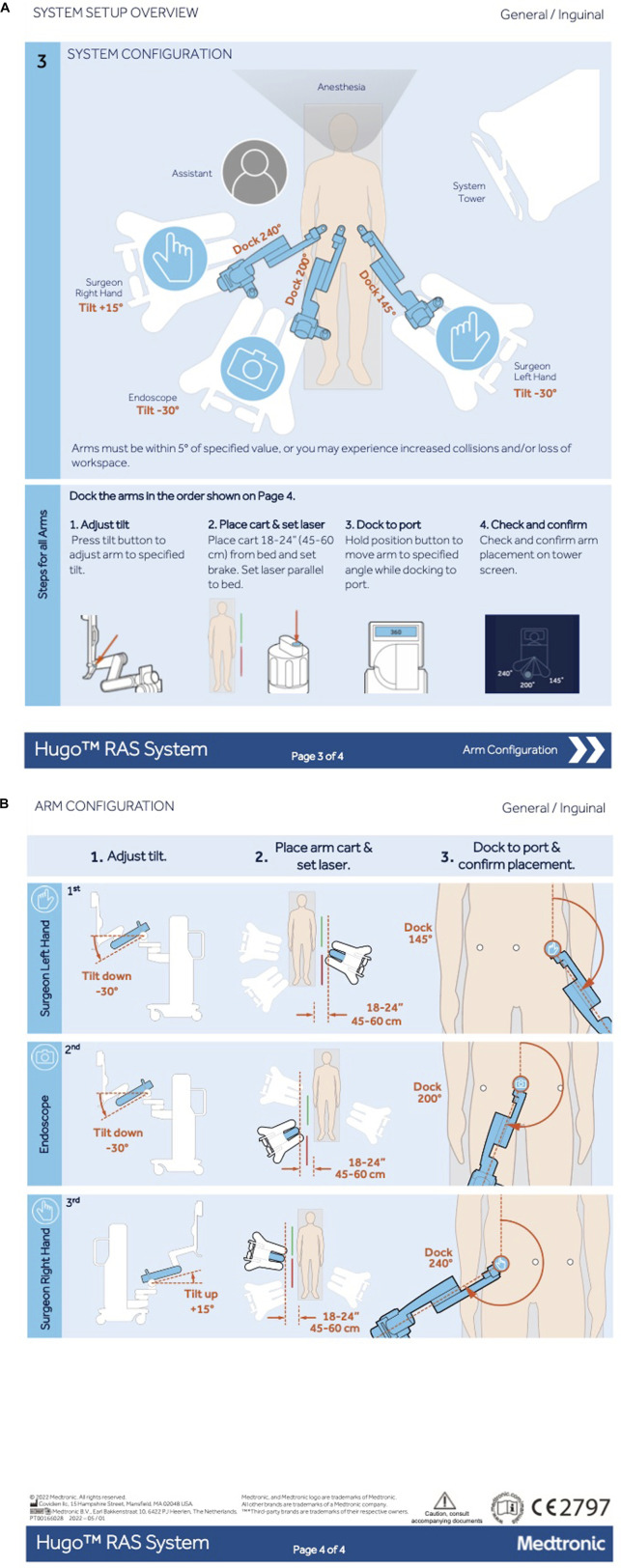
Set up guide of Hugo™ RAS for inguinal hernia repair. Reproduced with permission from Medtronic, 2024. **(A)** Schematic overview of cart location and arm setup. **(B)** Schematic overview of the arm configuration.

Patients were operated consecutively using Hugo^TM^ RAS. The Hugo^TM^ RAS is different from the da Vinci Xi in that it has an open console and a modular set up, with separate arm carts to be positioned around the patient. There is one arm cart for the camera endoscope and two arm carts for the instruments. The patient was positioned in a dorsal decubitus with a mild Trendelenburg position of 10° and the arms tucked alongside the patient’s body. A 12 mm optical trocar was placed at the supraumbilical position after creation of pneumoperitoneum using a Veress needle with an intra-abdominal pressure of 12 mmHg. Two additional trocars (8 mm) are placed bilaterally in the flank at the level of the umbilicus under direct vision. The distance between the lateral trocars and the umbilical trocar was 8 cm at minimum. After inspection of both groins, a decision was made to perform either a unilateral or a bilateral repair. Mesh and peritoneal closure suture were introduced into the peritoneal cavity. The meshes used in the study were 13 × 15 cm for unilateral hernias and 13 × 28 cm for bilateral hernias (Progrip™ Self-Fixating Mesh, Medtronic, Minneapolis, MN, US). The suture for closure of the peritoneum was a slowly absorbable barbed suture 15 cm in length for each side (V-Loc™ 90, Medtronic, Minneapolis, MN, US). The trocars were docked to the robotic arms after positioning the arm carts using the Hugo^TM^ RAS CE approved set-up guide for inguinal hernia repair with the endoscope at the umbilicus (Figures 1A,B). Tilt angles were installed correctly, and the docking angles were placed with a maximum allowed deviation of the recommended angle of −5° to +5°. The need for intraoperative adjustments of the angels or the position of the arm carts and robotic arms was documented on the case report form (CRF).

Robotic instruments used were a large needle driver, a bipolar fenestrated grasper and monopolar curved shears. Inguinal hernia repair was performed according to the standard surgical principles, with mesh placement after appropriate preperitoneal dissection and critical view of the myopectineal orifice, as described by Jorge Daes and Edward Felix [9]. The self-fixating mesh was appropriately positioned, and no additional fixation was utilized. After mesh placement, the peritoneum and subsequently the skin were closed.

For the da Vinci group the procedure was described in detail in the previous publication [8]. The surgical technique was standardized in both groups with two exceptions. Firstly, in the da Vinci group the robotic system involved the da Vinci Xi and secondly, bilateral hernias were repaired using two separate meshes. In the Hugo SUSHI cohort one mesh of 13 × 28 cm was used, a technique previously shown to be feasible and safe for bilateral repairs [10].

### Endpoints

Primary endpoint of the study was the skin-to-skin operative time defined as the time between first skin incision and the last skin suture. Secondary endpoint was the total operative time defined as the time between arrival of the patient in the operating room and the time the patient left the operating room. The adherence to the official set-up guide and the need for perioperative adjustments were documented. Patient reported outcome was documented using the EuraHS quality of life (QoL) instrument preoperatively and at the follow up clinical control visit at 4 weeks postoperatively [11].

### Variables and Data Measurement

Patient data recorded include: age; BMI; recurrent inguinal hernia; previous abdominal surgeries; unilateral versus bilateral hernia; hernia side in unilateral hernias; planned for day care clinic or overnight admission; preoperative and at 4 weeks postoperatively QoL assessed with the EuraHS QoL score [11]; inguinal hernia classification (medial-lateral-femoral, size 1-2-3) [1]; postoperative complications; length of stay; unplanned admission and overnight stay; complications at follow up at 4 weeks; seroma at 4 weeks, classified according to Morales et al [12].

During the course of the surgery, 8 time points were documented on the CRF: 1) arrival of the patient in the operating room, 2) end of induction of anaesthesia, 3) first skin incision, 4) surgeon into the console, 5) start placement of the mesh, 6) start suturing the peritoneum, 7) last skin suture, 8) patient leaving the operating room. These time points define seven distinct time intervals, T1-T7, in minutes. All data were entered in a REDCap^®^ database, downloaded to an excel file and double checked with the CRFs for errors before finalising the database and the start of the analysis. The total operative time was the sum of all 7 times intervals (T1+T2+T3+T4+T5+T6+T7). The skin-to-skin operative time was the sum of T3+T4+T5+T6.

### Study Size

A sample size of 50 patients was empirically chosen as being large enough to evaluate the learning curve effect on operative time and small enough to be performed within a reasonable time frame. It also mirrored the sample size of the da Vinci Xi group.

### Statistical Methods

The statistical methodology was selected and implemented by an independent statistician. Patient characteristics were summarized using proportions (%N) or by means and standard deviations (SD). Differences in continuous or dichotomous variables between groups were analysed using the T-test or Fisher’s exact test. Overall operative times were calculated for both study groups. They were also analysed separately for the first 25 and the last 25 patients in each group. The evolution of operative time during the learning curve was graphically illustrated using least square regression lines. Operative times for both study groups were also calculated overall and specifically for unilateral and bilateral inguinal hernia repairs. For the calculation of the EuraHS QoL scores (total and subdomains) the previously described methodology was used [13]. The distributions of these QoL scores were summarized both numerically and graphically, employing median and interquartile ranges (P25-P75), and were compared using the Mann-Whitney U test. A p-value of ≤ 0.05 was considered statistically significant. All analyses were performed with SAS software (release 9.4, Cary, NC, US).

## Results

### Patient & Hernia Characteristics

Between September 2023 and December 2024, the first 50 patients operated with the Hugo™ RAS at Sint Vincentius hospital, Deinze (Belgium) were enrolled in this study (Hugo SUSHI cohort). The da Vinci group consists of the first 50 patients who were operated with the da Vinci Xi during a 5-month inclusion period from September 2016 until January 2017 at Maria Middelares hospital, Ghent (Belgium).

Patient demographics, as summarized in Table 1, demonstrated no differences between groups in terms of gender, BMI, hernia recurrence rate and hernia side. The patients in the Hugo SUSHI cohort were older with a mean (SD) of 65.0 (14.2) years, compared to 58.7 (15.3) years in the da Vinci group (p = 0.035).

**TABLE 1:** Patient & hernia characteristics.

Characteristic	Hugo™ RAS n = 50	Da Vinci Xi n = 50
Patient: Age at operation (years) Gender, female Body Mass Index (kg/m^2^) Hernia: Recurrent Side Left Right Bilateral	65.0 (14.2) 8.0% (4/50) 25.3 (2.7) 6.0% (3/50) 24% (12/50) 28% (14/50) 48% (24/50)	58.7 (15.3) 2.0% (1/50) 25.4 (3.3) 6.0% (3/50) 32% (16/50) 36% (18/50) 32% (16/50)

### Perioperative Outcomes

Table 2 shows the perioperative outcomes of the Hugo SUSHI cohort and da Vinci control group. Two intraoperative complications occurred in the Hugo SUSHI cohort (4%). These complications were not procedure- or robot-related, consisting of one patient vomiting during induction of anaesthesia and one patient with an episode of atrial fibrillation. The setup guide developed for the Hugo RAS system for inguinal hernia repair (as shown in Figure 1) was validated and no adjustments needed to be made perioperatively. There were three instances of robot related issues during the operations. In all three cases, this did not lead to any harm to the patient but only to longer operation times. All errors could be solved during the operation. One error was a cart failure with rebooting taking 25 min, another error a start-up failure causing a 7 minute delay and one error was an instrument failure with no impact on the surgical time.

**TABLE 2:** Perioperative outcomes.

Outcome	Hugo™ RAS n = 50	Da Vinci Xi n = 50	Significance^a^
Intra-operative complications	4.0% (2/50)	0.0% (0/50)	P = 0.49
Intrahospital complications	6.0% (3/50)	10.0% (5/50)	P = 0.71
Day care clinic	80.0% (40/50)	68.0% (34/50)	P = 0.25
Seroma			P = 0.71
Type I	8.0% (4/50)	8.0% (4/50)	
Type II	2.0% (1/50)	4.0% (2/50)	
Type III	0.0% (0/50)	2.0% (1/50)	

^a^ According to the T-test or Fisher’s exact test.

There is no significant difference in intrahospital complications between both groups. The intrahospital complication in the Hugo SUSHI cohort (n = 3) were postoperative nausea and vomiting, haematuria and urinary retention. The haematuria was not considered procedure-related since the patient presented with kidney stones only days before the procedure and already had a ureteral stent *in situ*. All five patients in the da Vinci group who experienced an intrahospital complication had urinary retention. Most patients in both cohorts did not stay overnight. In the Hugo SUSHI cohort, 80% of patients were discharged the same day, compared to 68% in the Da Vinci group. Postoperative seroma occurred in 10% of patients in the Hugo SUSHI cohort, compared to 14% in the Da Vinci group.

### Operative Time

The detailed outcome data for the individual time blocks for both cohorts is presented in Table 3. All steps took less time in the Hugo SUSHI cohort, except for T6 (closure of the peritoneum).

**TABLE 3:** Time intervals.

Mean (SD) minutes		Hugo™ RAS n = 50	Da Vinci Xi n = 50
T1	Arrival in the operating room-End of induction of anaesthesia	9.0 (3.5)	14.3 (5.9)
T2	End of induction of anaesthesia-First skin incision	11.9 (3.9)	15.0 (6.0)
T3	First skin incision-Surgeon into the console	10.6 (4.7)	12.0 (4.2)
T4	Surgeon into the console-Introduction of the mesh	17.6 (6.4)	24.1 (9.8)
T5	Introduction of the mesh-Start of suturing the peritoneum	6.6 (2.6)	9.3 (5.1)
T6	Start of suturing the peritoneum-Last skin suture	22.3 (7.0)	17.5 (7.2)
T7	Last skin suture-Patient leaving the operating room	7.7 (3.2)	10.5 (5.0)

SD, standard deviation.

The outcome data for the total operative time and the skin-to-skin operative time is presented in Table 4. The operative times for unilateral and bilateral hernias separately and for the first 25 patients and the subsequent 25 patients for each group are mentioned. The mean total operative time with Hugo^TM^ RAS was 85.6 min and for da Vinci Xi 102.7 min. Operative times in the Hugo SUSHI cohort were significantly shorter with a mean difference of 17 min (95% CI +10.0 to +24.0, p < 0.001). This significant difference is not present for the skin-to-skin operative times, with 57.0 min in the Hugo SUSHI cohort and 62.8 min in the da Vinci group and a mean difference of 5.9 min (95% CI -0.9 to +12.6, p = 0.09).

**TABLE 4:** Total and skin-to-skin operative times of r-TAPP.

Mean (SD) minutes	Hugo™ RAS n = 50	Da Vinci Xi n = 50	Mean difference (95% CI), P-value*
Total operative time			
All patients Unilateral hernia** Bilateral hernia** First 25 patients Second 25 patients	85.6 (12.8) 80.0 (11.3) 91.8 (11.6) 88.2 (11.6) 83.0 (13.6)	102.7 (21.4) 94.0 (17.4) 121.0 (17.4) 110.6 (19.9) 94.7 (20.1)	−17.0 (−24.0 to −10.0), P < 0.001 −14.0 (−21.9 to −6.2), P < 0.001 −29.3 (−39.5 to −19.0), P < 0.001 −22.4 (−31.7 to −13.0), P < 0.001 −17.0 (−26.2 to −7.7), P = 0.021
Skin-to-skin operative time			
All patients Unilateral hernia** Bilateral hernia** First 25 patients Second 25 patients	57.0 (11.9) 51.1 (10.5) 63.3 (10.0) 57.1 (10.0) 56.9 (13.7)	62.8 (20.7) 54.4 (16.1) 80.8 (18.2) 74.0 (20.5) 51.6 (14.0)	−5.9 (+12.6 to +0.9), P = 0.09 −3.3 (−10.6 to +4.0), P = 0.37 −17.4 (−27.8 to −7.1), P < 0.001 −11.7 (−21.5 to −1.9), P < 0.001 +5.2 (−2.6 to +13.1), P = 0.19

SD, standard deviation; CI, confidence interval; *According to the T-test or Fisher’s exact test ** Note: da Vinci n = 16 bilateral vs n = 34 unilateral; Hugo RAS n = 24 bilateral vs n = 26 unilateral.

The skin-to-skin operative time for unilateral hernias with Hugo^TM^ RAS was not significantly different from the operative time with da Vinci Xi. The mean skin-to-skin operative time for bilateral hernias with Hugo^TM^ RAS was significantly shorter compared to da Vinci Xi, with a mean difference of 17.4 min (95% CI +7.1 to +27.8, p < 0.001). When comparing the first 25 patients of both groups, the mean total operative time and skin-to-skin operative time were significantly shorter in the Hugo SUSHI cohort, with a mean difference of resepectively. The difference in skin-to-skin operative time was no longer present when comparing the second 25 patients of each group.

### Learning Curve

**FIGURE 2:**
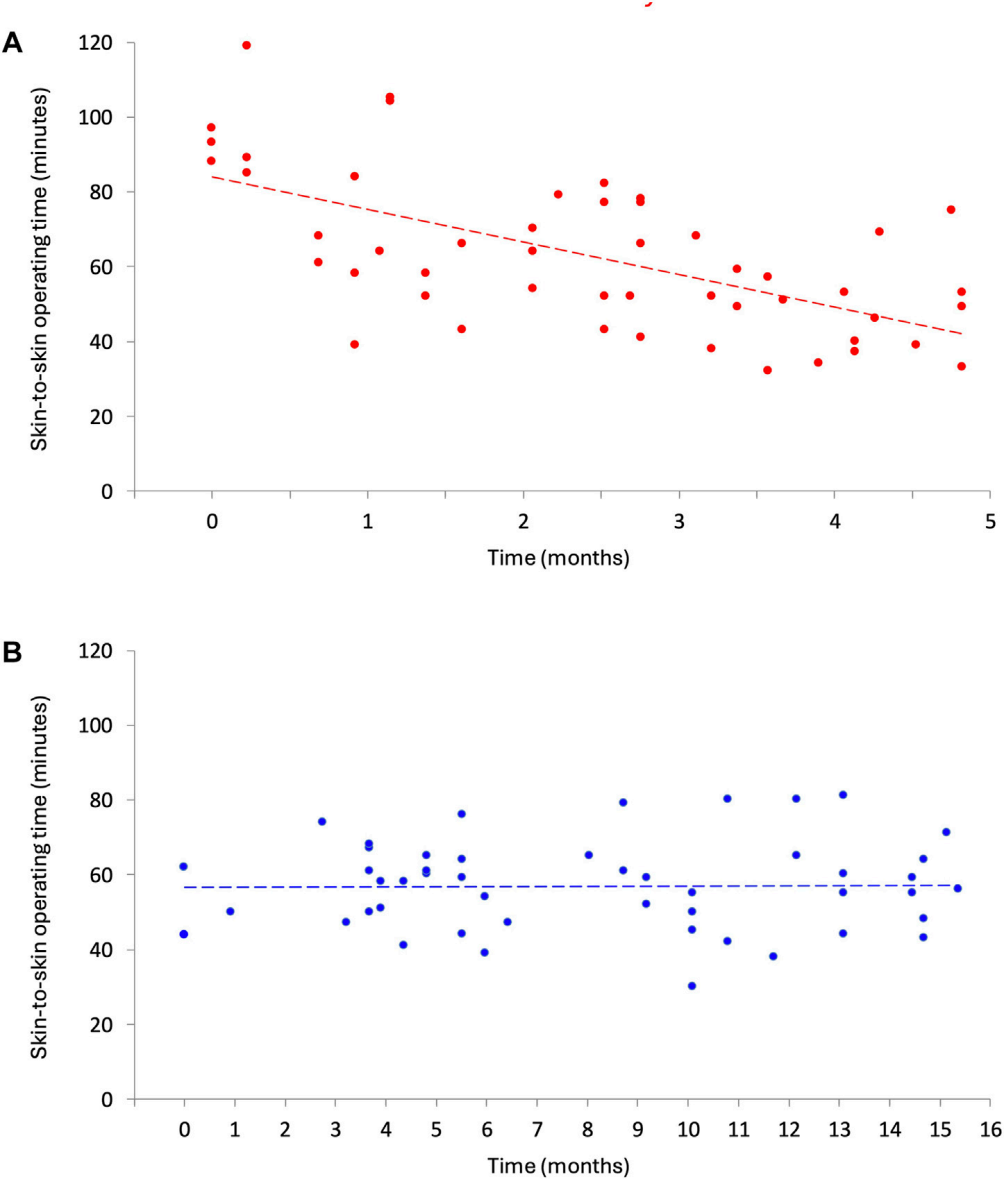
Change in skin-to-skin operative time for r-TAPP. **(A)** da Vinci Xi (sept 2016 - jan 2017). **(B)** Hugo^TM^ RAS (sept 2023 - dec 2024).

The evolution of the learning curve of the skin-to-skin operative time over time is graphically depicted for each cohort separately in Figures 2A,B. In the Hugo SUSHI cohort, the skin-to-skin operative time remained stable across cases, indicating no measurable learning curve.

### Quality of Life

**FIGURE 3:**
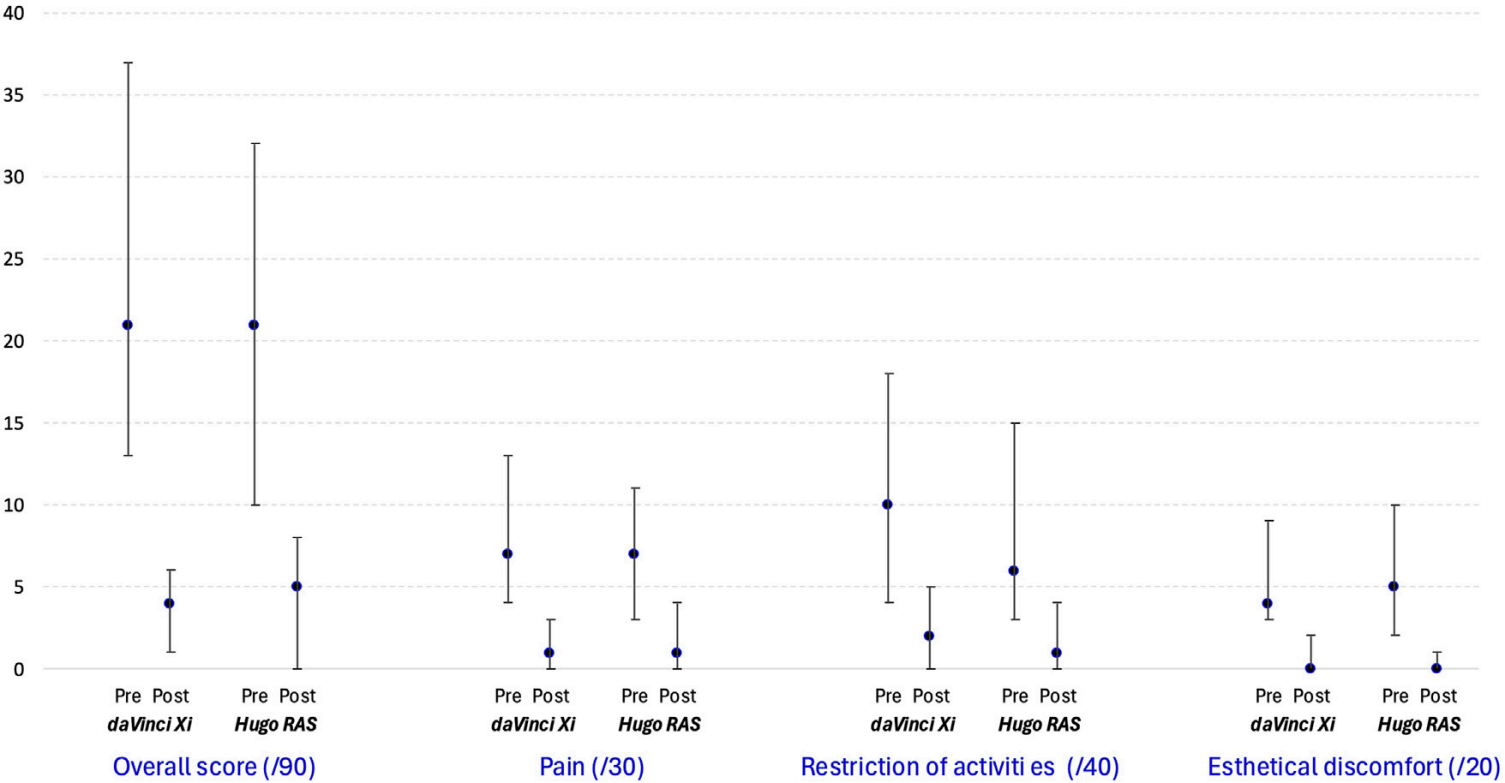
Median and interquartile boundary values (P25-P75) for measured EuraHS QoL score overall and per category.

The results of preoperative and postoperative analysis of the EuraHS QoL scores are presented in Table 5. There was no difference between the study groups overall, for the specific domains of the EuraHS QoL scores preoperatively and postoperatively, or for the change between preoperative and postoperative assessments. EuraHS QoL scores significantly improved from preoperative to postoperative assessment across all domains in both groups, which is graphically depicted in Figure 3.

**TABLE 5:** Measured EuraHS QoL score overall and per category preoperatively, 1-month postoperatively and change in score after r-TAPP.

Time interval (mean (SD) minutes)	Hugo™ RAS n = 50	Da Vinci Xi n = 50	Significance**
Preoperative	n = 50	n = 39	
Overall EuraHS QoL score	21 (10–32)	21 (13–37)	P = 0.55
“Pain” domain	7 (3–11)	7 (4–13)	P = 0.48
“Restriction of activities” domain	6 (3–15)	10 (4–18)	P = 0.18
“Esthetical discomfort” domain	5 (2–10)	4 (3–9)	P = 0.71
1-Month postoperative	n = 47	n = 48	
Overall EuraHS QoL score	5 (0–8)	4 (1–6)	P = 0.69
“Pain” domain	1 (0–4)	1 (0–3)	P = 0.60
“Restriction of activities” domain	1 (0–4)	2 (0–5)	P = 0.50
“Esthetical discomfort” domain	0 (0–1)	0 (0–2)	P = 0.08
Change from pre- to postoperative			
Overall EuraHS QoL score	−15.0 (−24 to −6) ***	−13.5 (−26 to −5.5) ***	P = 0.99
“Pain” domain	−4 (−8 to −1) ***	−5 (−7 to −1) ***	P = 0.98
“Restriction of activities” domain	−4 (−10 to 0) ***	−6 (−11 to 0) ***	P = 0.86
“Esthetical discomfort” domain	−4 (−8 to −2) ***	−3 (−8 to −0.5) ***	P = 0.24

*Median (P25-P75) ** According to Mann-Whitney U test *** All changes are significant at the P < 0.001 level.

## Discussion

This study is the first to compare the learning curves of the Hugo™ RAS and the da Vinci Xi robotic platforms for r-TAPP, using operative time as a surrogate marker of procedural efficiency.

The mean total operative time was significantly shorter with the Hugo™ RAS, whereas the mean skin-to-skin operative times did not differ significantly between the two groups. The longer total operative times observed in the da Vinci group may be attributed to differences in anaesthesiology teams and nursing staff between the two hospitals. It is important to consider that the procedures in the Hugo SUSHI cohort were performed several years later, during a period when robotic surgery had become more normalized in daily surgical practice. This broader institutional and team-based familiarity with robotic workflows may also have contributed to the shorter total operative times observed in this group. A more detailed analysis revealed a significant time advantage for the Hugo™ RAS in the initial 25 cases, which diminished in the subsequent cases. This seems to reflect the difference in experience of the surgeon during the adaption phase of the two systems.

Interestingly, the skin-to-skin operative times are comparable between the two groups, despite a higher proportion of bilateral hernias in the Hugo SUSHI cohort compared to the da Vinci group (48% vs. 32%). In the Hugo SUSHI cohort, the mean skin-to-skin time for bilateral hernias was 63.3 min, which is notably shorter than the 80.8 min in the da Vinci group. In a previously published study on bilateral TAPP using a single large mesh, procedures were performed laparoscopically by the same surgeon involved in the current study, with a reported mean skin-to-skin operative time of 76 min [10]. These findings suggest that robotic operative times for bilateral r-TAPP currently align to those achieved during the laparoscopic era.

The patients in the Hugo SUSHI cohort were significantly older, which can be explained by the difference between the two hospital sites where the study was done, with the Sint Vincentius hospital as a smaller local hospital serving an older population. There was no significant difference in hernia type or number of recurrent hernias. The complication rates were low and comparable between groups, with no procedure-related intraoperative complications. The incidence of asymptomatic seroma at 4-week follow-up did not differ meaningfully between platforms and is comparable with current literature [14]. Furthermore, the significant improvement in postoperative quality of life reinforces the overall benefit of minimal invasive approaches in inguinal hernia repair.

Our findings align with previous research supporting the feasibility and safety of the Hugo™ RAS for robotic-assisted abdominal and pelvic procedures [6]. A comparative studie of the same platforms for prostatectomy found a flat line in procedure time over time, but a decline with the Hugo™ RAS [15]. Our findings are particularly consistent with the early European experience reported by Ferri et al., who demonstrated successful implementation of the Hugo™ RAS for inguinal hernia repair [16]. By comparing the SUSHI cohort with the first 50 da Vinci Xi performed by the same surgeon, our study provides a unique opportunity to evaluate both systems during the early adoption phase. The surgeon’s extensive prior experience with the da Vinci system likely influenced the performance with the Hugo™ RAS. We expect novice robotic surgeons starting inguinal hernia repair with the Hugo™ RAS would experience a similar learning curve as shown in the da Vinci group.

Several important limitations should be acknowledged. This was a non-randomized comparison using prospectively collected data from two separate time periods and institutions, which may introduce selection and institutional bias. Also, only one surgeon performed the procedures which limits generalizability. Lastly, while operative time is a useful surrogate for procedural efficiency, it does not capture all aspects of surgical quality, such as ergonomics, long-term outcomes, or cost-effectiveness. However, this was beyond the scope of this study.

## Conclusion

Transitioning from the da Vinci Xi to Hugo™ RAS for r-TAPP was safe and efficient for an experienced robotic surgeon. The absence of a measurable learning curve in skin-to-skin operative time, supports the feasibility of adopting the Hugo™ RAS without compromising operative performance.

## Data Availability

The raw data supporting the conclusions of this article will be made available by the authors, without undue reservation.
